# Case Report: Spontaneous Rupture of Inferior Epigastric Artery Masquerading as Inguinal Hernia

**DOI:** 10.5811/cpcem.2020.9.48629

**Published:** 2020-10-19

**Authors:** Kathryn Sulkowski, Christian Young

**Affiliations:** University of Nevada, Department of Emergency Medicine, Las Vegas, Nevada

**Keywords:** inferior epigastric artery rupture

## Abstract

**Introduction:**

Spontaneous rupture of an inferior epigastric artery aneurysm is rare with very few cases reported in the medical literature. Although surgical options are available, this case was managed conservatively with outpatient management.

**Case Report:**

A 29-year-old male presented with right groin pain and swelling that was initially felt to be consistent with an incarcerated inguinal hernia. Further evaluation revealed spontaneous rupture of an inferior epigastric artery aneurysm. The patient was treated conservatively and was ultimately discharged home from the emergency department.

**Conclusion:**

Due to the similar clinical presentations, it was important to consider a broad differential to ultimately arrive at the correct diagnosis. In some reported cases of spontaneous epigastric artery aneurysm, surgical intervention was required for control of the bleeding. In our patient, however, conservative management was employed, and the patient was able to be safely discharged with close outpatient follow-up.

## INTRODUCTION

Spontaneous rupture of an inferior epigastric artery aneurysm is very rare with very few cases reported in the medical literature.^1^,^2^,^4^,^5^ Presentations are typically varied and with symptoms that are commonly mistaken for a more common diagnosis, making the definitive diagnosis difficult to come to unless there is a clear provocation, such as obvious trauma.^1^ In this report we describe the case of an inferior epigastric artery aneurysm rupture presenting as right inguinal pain occurring shortly after sexual intercourse. The symptoms were initially felt to be due to an incarcerated inguinal hernia. Although surgical options are available, this case was managed conservatively with outpatient management.

## CASE REPORT

A 29-year-old male presented to the emergency department (ED) with progressive right groin pain and swelling for the prior 24 hours. The patient stated that while he was incarcerated, he was informed by medical staff that he had an inguinal hernia. The patient was released from prison to a sober living facility the day prior to presentation to the ED. He reported a history of sexual intercourse shortly before symptom onset. Since that time, he had noted increased swelling and tenderness to the area. He presented to the ED due to the increasing severity of the pain. He had a normal bowel movement one day prior to presentation and endorsed passing gas the day of presentation. He denied nausea, vomiting, diarrhea, fever, chest pain, or shortness of breath. He had no dysuria, hematuria, or flank pain. He denied any associated penile discharge, testicular pain, or skin rashes or lesions.

The patient had no past medical history and reported no daily medications. He was newly sexually active with one female partner one time prior to presentation and had been abstinent in prison. He denied a history of sexually transmitted infections. He smoked cigarettes and drank alcohol occasionally but denied any illegal drug use.

Physical exam revealed a thin male resting comfortably on the stretcher. Vital signs were only notable for mild resting tachycardia with a pulse of 102 beats per minute; blood pressure 125/73 millimeters mercury; oral temperature 37.1ºC; and oxygen saturation 100% on room air. The head, eyes, ears, nose, and throat, cardiac, and respiratory exams were all unremarkable. The abdomen was soft, nontender, nondistended, with normoactive bowel sounds and no organomegaly. There was no costovertebral angle tenderness.

Genitourinary exam demonstrated a large mass in the right groin region with no overlying skin changes. The bulge was mildly tender to palpation, with no fluctuance noted. Gentle pressure applied to the area did not result in reduction of the bulge. The testicular exam demonstrated normal testicular lie with no abnormal swelling, tenderness, discoloration, or rash. The cremasteric reflex was present bilaterally. The penis was nontender, uncircumcised, and had no lesions or rashes.

Initial diagnostic evaluation demonstrated a white blood cell count of 10,740 cells per/liter (L) (reference [ref] range: 4,500 to 11,000 cells/L); hemoglobin of 15.3 grams per deciliter (g/dL) (ref range: 13.5–17.5 g/dL), and platelets of 233,000 cells/L (ref range: 150,000–450,000 cells/L). The comprehensive metabolic panel was only remarkable for an alanine aminotransferase of 61 international units (IU)/L (ref range: 29–33 IU/L); all electrolytes were within normal limits. A urinalysis demonstrated no cells or bacteria. Lactic acid was normal at 1.10 millimoles (mmol)/L (ref range: 0.5–2.2 mmol/L). The patient was administered 4 milligrams of morphine for pain, which improved his discomfort.

A computed tomography (CT) of the abdomen and pelvis was obtained out of concern for an incarcerated inguinal hernia. It revealed a heterogeneous mass-like process noted within the right inguinal region estimated at approximately 6.2 × 7.1 × 7.7 centimeters (cm) in size (Image). There was stranding of the adjacent fat of the groin with findings suggestive of large hematoma or heterogeneous, soft-tissue mass. There were also prominent lymph nodes and stranding of the fat seen within the right groin with largest lymph node estimated up to 1.8 × 0.7 cm in size. The mass was noted to extend to the musculature of the right groin but did not appear to be related to a hernia. Due to the unusual finding on the CT and the patient’s continued pain, general surgery service was consulted. After a bedside evaluation, it was determined that these findings were consistent with a spontaneous rupture of an inferior epigastric artery aneurysm. Per the general surgery team there was no need for admission or surgery. The patient was then discharged with instructions to use warm compresses to help the mass decrease in size over time and to use acetaminophen for pain control. At the time of discharge the patient was no longer tachycardic, was afebrile, and had a normal blood pressure. He did not present to our hospital or outpatient clinics again since his evaluation in the ED.

CPC-EM Capsule

What do we already know about this clinical entity?

*Almost no cases in the literature since the early 1900s have reported conservative management for spontaneous epigastric artery aneurysm*.

What makes this presentation of disease reportable?

*Though surgical management for bleeding is more common, outpatient management is an option in certain cases and saves the patient a potentially unnecessary surgery*.

What is the major learning point?

*In patients who present with sudden onset of groin pain and swelling, it is important to consider this diagnosis and the need for surgical versus conservative management*.

How might this improve emergency medicine practice?

*Physicians should keep a broad differential for groin pain and swelling while introducing a rare entity that may initially be thought to be an incarcerated hernia*.

## DISCUSSION

The rupture of inferior epigastric vessels is not a common occurrence. An exploration of both emergency medicine and surgical literature did not demonstrate a consistent explanation or description of this condition.^1^,^2^,^4^,^5^ The differential diagnosis for this presentation is diverse, and the outcomes/management vary based on which diagnosis is selected. While it is an uncommon presentation that can be easily confused with other diagnoses, early diagnosis will result in a decrease in unnecessary laboratory procedures and possibly even surgical intervention.^1^ Spontaneous rupture of an epigastric artery aneurysm typically presents with sudden onset of severe pain to the left or right of midline, typically at the level of the umbilicus but it could also be lower, even in the groin.^1^ Although typically abrupt in onset, several cases have been reported that involved insidious onset over five to seven days.^1^

The first case of inferior epigastric artery rupture was reported in 1857 by Richardson.^2^ After this there were a few case reports in the literature in the early 1900s. Recently there have been a few more case reports detailing catastrophic cases of rupture in the setting of aortic dissection.^3^ Typically these cases are treated with surgery, traditionally open repair, and more recently with endovascular technology. Depending on the location in the artery where the aneurysm and rupture occur dictates the need for surgery as those at the distal branches tend to cause less catastrophic bleeding, which typically tamponades off in the tissues.^1^

As there is a long list of diagnoses for groin or abdominal pain with a visible bulge or mass, many conditions can be confused for the rupture of the inferior epigastric artery aneurysm. Some examples are incarcerated or strangulated inguinal hernia, tumors of the abdominal musculature, ovarian cysts, appendicitis, abscess of the abdominal wall, mesenteric thrombosis, muscle rupture and, in a few reported cases, intestinal obstruction.^1^, ^4^ Various physical exam clues can help to delineate this diagnosis from others on the list. One characteristic is that the mass does not change its position and always appears fixed in the abdominal wall. This mass will remain present regardless of the patient’s positioning and cannot be moved from side to side or reduced with pressure on the area. This sign was first described by Fothergill.^5^ Some cases describe extreme pain and, in rare cases, frank shock. However, most cases described discuss slight elevations in temperature, pulse, and white blood cell count.^5^

With regard to treatment options, there are two schools of thought. The majority of surgeons appear to advocate for surgical intervention to find and ligate the bleeding as the safest and surest therapy.^5^ However, there is a smaller minority that advocates for a more conservative non-operative approach. This includes warm compresses, pain control, and abdominal compressive devices.^1^ The conservative approach does run the risk of continued and worsening bleeding, abscess formation, or calcification with chronic pain.^1^ Conservative management in a select patient population, however, can prevent unnecessary surgical intervention. In our patient, with stable vital signs, pain that was adequately controlled, with no signs of continued bleeding (the mass had not increased in size in the nearly eight hours he was in the ED), and a patient who understood the reasons to return to the ED, discharge with symptomatic treatment proved the less-invasive option.

## CONCLUSION

We report the case of a 29-year-old male with a ruptured inferior epigastric artery aneurysm. He presented to the ED with a painful right groin bulge, which is a typical presentation of this entity. CT demonstrated a mass concerning for a hematoma but no signs of hernia. Spontaneous rupture of an inferior epigastric artery aneurysm is a rare entity that can masquerade as multiple other diagnoses. In patients who present to the ED with sudden onset of new groin pain and swelling, it is important to consider this diagnosis and consider early consultation with a surgical team, especially in the case of questionably stable patients who will likely need to go to the operating room for stabilization. In stable patients, however, conservative management can be appropriate.

## Figures and Tables

**Image:**
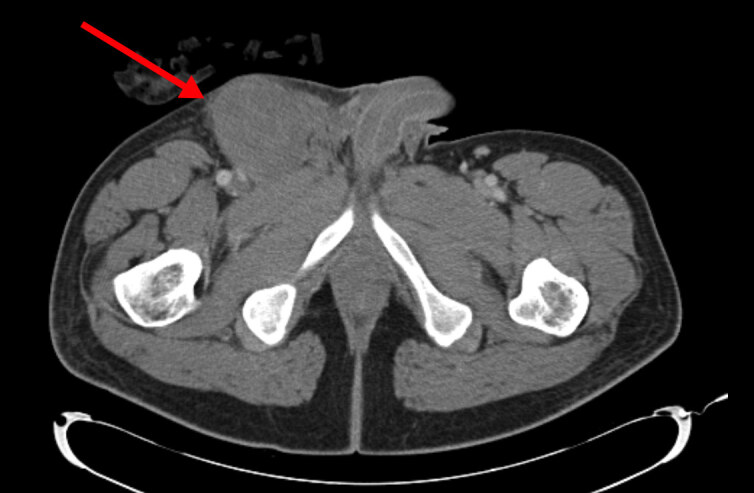
Computed tomography demonstrating a large heterogeneous, mass-like process within the right groin, which may be related to an unusually large hematoma.

## References

[1] Payne RL (1938). Spontaneous rupture of the superior and inferior epigastric arteries within the rectus abdominis sheath. Ann Surg.

[2] Herrman C (1946). Rupture of the deep epigastric vessels. Am J Surg.

[3] Gou M, Feng X, Lu Q (2009). Endovascular covered stent for inferior epigastric artery rupture after EVAR for Stanford B aortic dissection. Euro J Vasc Endovasc Surg.

[4] Kinder CH (1952). A case of spontaneous rupture of the inferior epigastric artery simulating acute intestinal obstruction. Br J Surg.

[5] Murray SD, Burger RE (1954). Rupture of the inferior epigastric vessels. Ann Surg.

